# Effects of electromyostimulation on sarcolipin and Ca^2+^ signaling in rat skeletal muscles

**DOI:** 10.1016/j.bbrep.2026.102588

**Published:** 2026-04-21

**Authors:** Koki Okumura, Junya Takegaki, Kohei Sase, Naoki Fukao, Satoshi Fujita

**Affiliations:** a Graduate School of Sport and Health Science, Ritsumeikan University, 1-1-1 Noji-higashi, Kusatsu, Shiga, 525-8577, Japan; b Graduate School of Agricultural Science, Kobe University, 1-1 Rokkodai-cho, Nada-ku, Kobe, Hyogo, 657-8501, Japan; c Faculty of Health and Sports Sciences, Toyo University, 1-7-11 Akabanedai, Kita-ku, Tokyo, 115-8650, Japan

**Keywords:** Resistance training, Sarcolipin, Sarcoplasmic reticulum Ca^2+^-ATPase (SERCA), Calcineurin, Regulator of calcineurin 1 (RCAN1), Ca^2+^/calmodulin-dependent protein kinase II (CaMKII)

## Abstract

Calcium ion (Ca^2+^) acts as a second messenger involved in various adaptations by activating signaling pathways. The expression of Ca^2+^ regulators is modified by resistance training. Thus, Ca^2+^ signaling may be altered during a period of resistance training. In this study, male Sprague-Dawley rats underwent repeated resistance exercise via transcutaneous electromyostimulation, and the expression of Ca^2+^-related factors was examined 3 h after the 1st, 5th, and 10th exercise sessions. Expression of sarcolipin (SLN) and regulator of calcineurin 1 (RCAN1) was significantly higher in exercised legs than control legs after the 5th (*p* < 0.001) and 10th sessions (*p* < 0.001). Calcineurin expression showed significant main effects of exercise (*p* = 0.009) and session (*p* < 0.001). A significant main effect of session was also observed for Ca^2+^/calmodulin-dependent protein kinase II (CaMKII) (*p* < 0.001), CaMKII α (*p* < 0.046), CaMKII β (*p* = 0.002), and CaMKII γ (*p* < 0.001). In contrast, expression of sarcoplasmic reticulum Ca^2+^-ATPase (SERCA) did not change with repeated electromyostimulation. Pearson's correlation analysis showed a significant positive correlation between the expression of SLN and RCAN1 (*r* = 0.630, *p* = 0.005) and between SLN and CaMKII γ (*r* = 0.471, *p* = 0.048), as well as a trend for a positive correlation between SLN and CaMKII (*r* = 0.463, *p* = 0.053). These results suggest that repeated sessions of electromyostimulation increase SLN expression, which may in turn contribute to enhanced Ca^2+^ signaling after exercise.

## Highlights

• Repeated electromyostimulation elevates sarcolipin in rats, peaking by session 5.
• Sarcolipin upregulation correlates with RCAN1 and CaMKIIγ, indicating enhanced Ca^2+^ signaling.
• SERCA protein level remains unchanged across the training sessions.

## 1 Introduction

Skeletal muscle undergoes a variety of changes in response to exercise. Among these, resistance training induces well-characterized adaptations, including increased muscle mass and strength, a shift in muscle fiber types (i.e., type Ⅱx to type Ⅱa), greater mitochondrial content and turnover, and improved glucose metabolism [[1], [2], [3], [4]]. Such training-induced adaptations require the accumulation of repeated acute responses, but the precise mechanisms underlying the effects of repeated bouts of exercise remain incompletely understood.

Growing evidence suggests that calcium ion (Ca^2+^) flux affects muscle mass, muscle fiber types, mitochondrial content and turnover, and glucose metabolism processes [[5], [6], [7], [8], [9], [10], [11]]. Besides, it has been reported that activation of Ca^2+^ signaling is induced by increased cytosolic Ca^2+^ concentration and contributes to muscle fiber type shifts, increased mitochondrial biogenesis, and increased glucose metabolism [[8], [9], [10], [11], [12], [13], [14]]. Resistance exercise is an exercise mode that changes the intense Ca^2+^ concentration in skeletal muscle [15]. However, Ca^2+^ metabolism during the training period is unknown. Thus, clarifying how Ca^2+^ metabolism is regulated by repeated resistance exercise could shed new light on the cellular events that occur during the training period, which could contribute to a better understanding of how skeletal muscle adaptation can be accelerated.

Sarcolipin (SLN) is a protein consisting of a 31-amino acids abundantly expressed in muscle that is involved in regulating cytosolic Ca^2+^ concentration by inhibiting the activity of sarcoplasmic reticulum Ca^2+^-ATPase (SERCA) [[16], [17], [18], [19]]. SLN expression is promoted by calcineurin and suppressed by the regulator of calcineurin 1 (RCAN1, also known as DSCR1 or MCIP1), an endogenous inhibitor of calcineurin [20,21]. Previous studies have reported that SLN expression in the vastus lateralis muscle increases after 8 weeks of low-load resistance training in physically active humans [22] and that high *Sln* mRNA expression and SLN protein expression are observed in a 2-week overload mouse model [23,24]. These findings suggest that SLN expression may be upregulated by chronic mechanical stress in skeletal muscles. However, it remains unclear how SLN expression and its regulators change in response to repeated resistance exercise. It has been reported that SLN expression levels are affected by exercise, dietary habits, and genetics [22,[25], [26], [27]]. Therefore, in this study, we used rats to minimize these potential confounding factors, enabling us to clarify the initial response and the changes in response over time during the training period.

It has been reported that increased SLN expression raises cytosolic Ca^2+^ concentration and activates the Ca^2+^ signaling factors calcineurin and Ca^2+^/calmodulin-dependent protein kinase II (CaMKII), thereby promoting mitochondrial biogenesis [8,11,13]. Mice overexpressing SLN exhibit increased running capacity and attenuated fatigue-induced declines in force output of the extensor digitorum longus and soleus muscles [28]. In contrast, the overloaded plantaris muscle of Sln ^−/−^ mice does not show activated calcineurin signaling, increased proportion of type I and type IIa fibers, or increased muscle fiber cross-sectional area compared with wild-type mice [24]. Therefore, SLN expression may affect exercise performance and muscle phenotype, which are adaptations associated with Ca^2+^ signaling. However, the relationship between SLN expression and Ca^2+^ signaling factors during repeated resistance exercise remains unclear.

The purpose of this study was to clarify how Ca^2+^ regulatory and signaling factors in skeletal muscle change in response to repeated resistance exercise. To do this, we induced muscle contractions with transcutaneous electrical stimulation to simulate high-intensity resistance exercise.

## 2 Materials and methods

### 2.1 Animals and experimental design

**Fig. 1:**
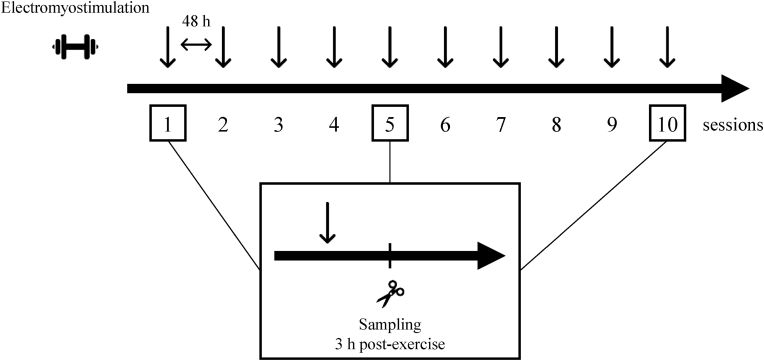
Experimental design. Electromyostimulation was applied to the right leg of rats every 48 h to simulate resistance exercise. Rats were sacrificed 3 h after the end of the 1st, 5th, or 10th sessions.

Eighteen male Sprague-Dawley rats (10 weeks old) were purchased from Shimizu Laboratory Supplies (Kyoto, Japan). All animals were housed at 23 ± 1 °C under a 12-h/12-h light-dark cycle and were provided food and water ad libitum. After a week of environmental acclimation, we randomly divided the rats into 1st, 5th, and 10th session groups. We simulated resistance exercise using electromyostimulation, which was applied to the right leg every 48 h (Exercise), while the left leg was not stimulated and served as a resting control (Control). Rats were fasted overnight before muscle collection. Rats were then anesthetized and exsanguinated 3 h after the end of the 1st, 5th, or 10th sessions, and the gastrocnemius muscles were collected (Fig. 1). The muscles were frozen in liquid nitrogen and stored at −80 °C until analysis. This study was approved by the Ethics Committee for Animal Experiments at Ritsumeikan University (BKC2018-037).

### 2.2 Electrical stimulation protocol simulating resistance exercise

Electrical stimulation simulating resistance exercise was carried out according to the protocol described in the previous study [29]. Briefly, Rats were anesthetized with isoflurane (095-06573, FUJIFILM Wako Pure Chemical, Osaka, Japan), and both legs were shaved with a razor and ethanol. Rats were placed in a prone position on the flat stand, the knees were fixed, and the ankles were fixed at 90°. Subsequently, an electrode pad (G-271A, NIHON KOHDEN, Tokyo, Japan) cut to 10 mm by 5 mm was attached to the position on the gastrocnemius muscle of the right leg. Electrical stimulation was then applied to the gastrocnemius muscle using an electrical stimulator (SEN-8203, NIHON KOHDEN) and an isolator (SS-104J, NIHON KOHDEN). Electrical stimulation was performed at a voltage of ∼30 V and a frequency of 100 Hz to induce maximal isometric muscle contraction. Each set consisted of 10 contractions (3-s stimulation and 7-s relaxation), with 3 min of rest between sets. A total of 5 sets were performed [30]. This protocol is considered a suitable model of resistance exercise, since previous studies have confirmed that it activates the mechanistic target of rapamycin (mTOR) signaling and increases muscle protein synthesis as assessed by the SUnSET method [[29], [30], [31]]. Additionally, this model induces maximal isometric muscle contraction, allowing for standardized relative loading across animals.

### 2.3 Western blot analysis

Frozen muscle samples were crushed and powdered. The powder was mixed in RIPA buffer (9806S, Cell Signaling Technology, Danvers, MA, USA) containing a phosphatase inhibitor tablet (04906837001, Roche Diagnostics Deutschland GmbH, Mannheim, Germany) and a protease inhibitor tablet (11836170001, Roche Diagnostics Deutschland GmbH) on ice, and was homogenized. After the mixture was centrifuged (4 °C, 10000 g, 10 min) to collect the supernatant, the protein concentration of each muscle sample was measured using a protein assay kit (295-78401, FUJIFILM Wako Pure Chemical). The concentration of each muscle sample solution was adjusted to 2 mg/mL by mixing it with RIPA buffer, Blue Loading buffer (56036, Cell Signaling Technology), DTT (14265, Cell Signaling Technology), and distilled water. The adjusted sample solution was then boiled at 95 °C for 5 min. Protein (5-10 μg) was loaded into each well of a 7.5% or 10% TGX gel (1610173, Bio-Rad Laboratories, Hercules, CA, USA) and separated using electrophoresis. Samples were then transferred to a PVDF membrane (1620177, Bio-Rad Laboratories) and stained using Ponceau S to verify equal protein loading and for normalization.

The membranes were then blocked for 1 h with Tris-buffered saline with Tween 20 (TBST) containing 5% skim milk. After blocking, the membranes were washed with TBST and incubated with primary antibodies diluted in TBST containing 5% bovine serum albumin overnight at 4 °C. The primary antibodies were SLN (ABT13, MilliporeSigma, Burlington, MA, USA, 1:1000), SERCA1 (MA3-912, Thermo Fisher Scientific, Waltham, MA, USA, 1:40000), SERCA2 (MA3-919, Thermo Fisher Scientific, 1:1000), calcineurin (2614, Cell Signaling Technology, 1:5000), RCAN1 (sc-377507, Santa Cruz Biotechnology, Dallas, TX, USA, 1:5000), phosphorylated CaMKII (*p*-CaMKII) Thr287 (12716, Cell Signaling Technology, 1:5000), total CaMKII (t-CaMKII) (4436, Cell Signaling Technology, 1:5000).

After primary antibody incubation, the membranes were washed with TBST and incubated with secondary antibodies diluted in TBST with 3% skim milk for 1 h at room temperature. The secondary antibodies were HRP-linked anti-rabbit IgG (7074, Cell Signaling Technology, 1:2000) and HRP-linked anti-mouse IgG (7076, Cell Signaling Technology, 1:2000). After washing with TBST, chemiluminescence detection was performed using Immobilon Forte Western HRP Substrate (WBLUF0500, Merck Millipore, Burlington, MA, USA), and bands were visualized using the Vilber Bio Imaging FUSION system (FX7.EDGE, M&S Instruments, Osaka, Japan). Band intensity was quantified using ImageJ software v1.53a (National Institutes of Health, Bethesda, MD, USA). These SDS-PAGE and Western blot protocols were based on a previous study [32].

### 2.4 Statistical analysis

Comparisons were performed using two-way analysis of variance with exercise (Control, Exercise) and session (1st, 5th, 10th) as main effects. Multiple comparison tests were performed using the Bonferroni method when interactions were observed. Pearson's correlation analysis was performed on exercised legs to examine the relationships between SLN expression and Ca^2+^ signaling factors under repeated resistance exercise. Data are expressed as mean ± standard error. Statistical analysis was performed using IBM SPSS Statistics (Ver. 29; IBM Corp., Armonk, NY, USA), and statistical significance was set at *p* < 0.05.

## 3 Results

**Fig. 2:**
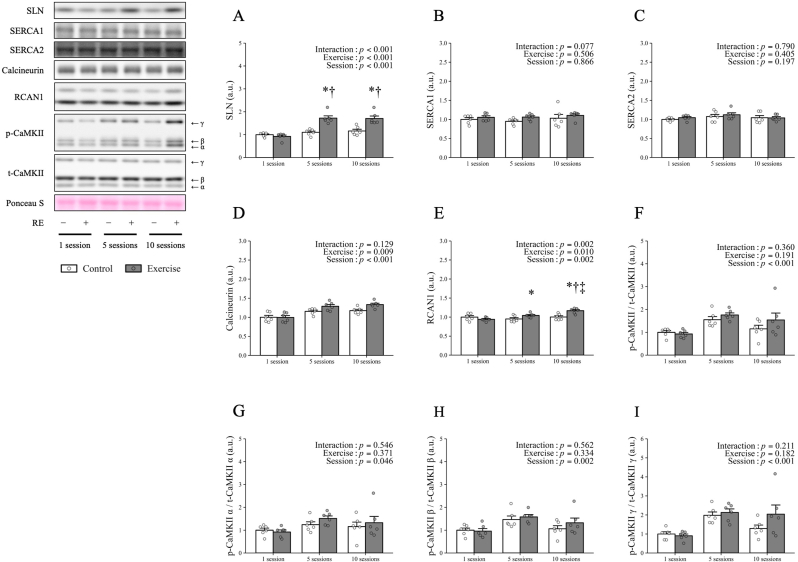
SLN, SERCA, SLN regulator factors, and Ca^2+^ signaling factors expression in rat skeletal muscle in response to repeated resistance exercise. (A) SLN expression. (B) SERCA1 expression. (C) SERCA2 expression. (D) Calcineurin expression. (E) RCAN1 expression. (F) Phosphorylated CaMKII. (G) Phosphorylated CaMKII α. (H) Phosphorylated CaMKII β. (I) Phosphorylated CaMKII γ. Representative bands are indicated on the left. The white bar and white plots indicate the control legs, the gray bar and gray plots indicate the exercised legs. a.u., arbitrary unit; CaMKII, Ca^2+^/calmodulin-dependent protein kinase II; *p*-CaMKII, phosphorylated CaMKII; RCAN1, regulator of calcineurin 1; RE, resistance exercise; SERCA, sarcoplasmic reticulum Ca^2+^-ATPase; SLN, sarcolipin; t-CaMKII, total CaMKII. The figure was created using RStudio (Version 2023.06.1 + 524, Posit PBC, Boston, MA, USA). Data are expressed as mean ± SE relative to the first session in control legs. *n* = 6 legs per condition. ∗: *p* < 0.05 vs. control legs for each session, †: *p* < 0.05 vs. 1st session in exercised legs, ‡: *p* < 0.05 vs. 5th session in exercised legs.

Changes in SLN, SERCA, calcineurin, RCAN1 expression, activation of CaMKII and its isoforms are shown in Fig. 2. SLN expression was significantly higher in exercised legs than control legs after the 5th (*p* < 0.001) and 10th (*p* < 0.001) sessions. In addition, SLN expression in exercised legs was significantly higher after the 5th (*p* < 0.001) and 10th (*p* < 0.001) sessions than the 1st session. However, no significant difference was observed between the 5th and 10th sessions in exercised legs (Fig. 2A). No significant differences in SERCA1 or SERCA2 expression were observed between control and exercised legs or between sessions (Fig. 2B and C). Although no interaction was observed for calcineurin expression, significant main effects of exercise (*p* = 0.009) and session (*p* < 0.001) were observed (Fig. 2D). RCAN1 expression was significantly higher in exercised legs than control legs after the 5th (*p* = 0.034) and 10th (*p* < 0.001) sessions. In addition, RCAN1 expression in exercised legs was significantly higher after the 10th session than after the 1st (*p* < 0.001) and 5th (*p* = 0.018) sessions (Fig. 2E). Although no interactions of CaMKII activation were observed, a significant main effect of session was observed (*p*-CaMKII/t-CaMKII: *p* < 0.001, *p*-CaMKII α/t-CaMKII α: *p* = 0.046, *p*-CaMKII β/t-CaMKII β: *p* = 0.002, *p*-CaMKII γ/t-CaMKII γ: *p* < 0.001) (Fig. 2F–I).

**Fig. 3:**
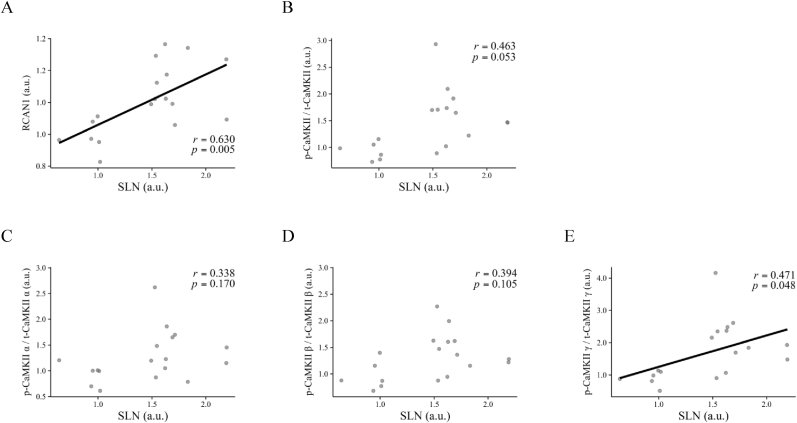
Correlation between SLN and Ca^2+^ signaling factors in response to repeated resistance exercise. Relationships between SLN expression and (A) RCAN1 expression, (B) phosphorylated CaMKII, (C) phosphorylated CaMKII α, (D) phosphorylated CaMKII β, and (E) phosphorylated CaMKII γ. CaMKII, Ca^2+^/calmodulin-dependent protein kinase II; *p*-CaMKII, phosphorylated CaMKII; RCAN1, regulator of calcineurin 1; SLN, sarcolipin; t-CaMKII, total CaMKII. The figure was created using RStudio (Version 2023.06.1 + 524, Posit PBC). Data includes exercised legs only.

The relationships between SLN and Ca^2+^ signaling factors after repeated electromyostimulation are shown in Fig. 3. There was a significant positive correlation between SLN and RCAN1 (*r* = 0.630, *p* = 0.005) (Fig. 3A). There was a trend for a positive correlation between SLN and *p*-CaMKII/t-CaMKII (*r* = 0.463, *p* = 0.053) (Fig. 3B). There was no significant correlation between SLN and *p*-CaMKII α/t-CaMKII α (*r* = 0.338, *p* = 0.170) (Fig. 3C) or *p*-CaMKII β/t-CaMKII β (*r* = 0.394, *p* = 0.105) (Fig. 3D). However, there was a significant positive correlation between SLN and *p*-CaMKII γ/t-CaMKII γ (*r* = 0.471, *p* = 0.048) (Fig. 3E).

## 4 Discussion

**Fig. 4:**
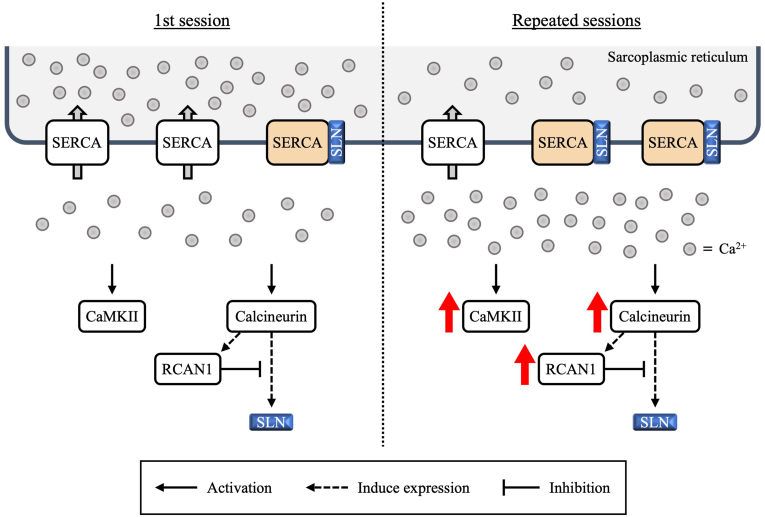
Suggested model of changes in Ca^2+^ regulators and Ca^2+^ signaling factors due to repeated resistance exercise. The results of this study suggest that repeated resistance exercise induces SLN expression, leading to an increase in SLN expression levels. Since SLN inhibits SERCA function, it may have influenced changes in cytosolic Ca^2+^ concentration [[16], [17], [18], [19]]. Furthermore, the increased SLN expression levels are suggested to be associated with enhanced activation of Ca^2+^ signaling factors 3 h after exercise. CaMKII, Ca^2+^/calmodulin-dependent protein kinase II; RCAN1, regulator of calcineurin 1; SERCA, sarcoplasmic reticulum Ca^2+^-ATPase; SLN, sarcolipin.

In this study, we collected gastrocnemius muscles 3 h after the 1st, 5th, and 10th sessions of electromyostimulation to investigate changes in skeletal muscle Ca^2+^ regulatory and signaling factors during repeated resistance exercise. SLN expression did not change after the 1st session but increased after multiple sessions. SLN expression was significantly positively correlated with RCAN1 and CaMKII γ and trended toward positive correlation with CaMKII. These results suggest that repeated electromyostimulation increases SLN expression and that increased SLN expression may be related to enhanced Ca^2+^ signaling after exercise (Fig. 4). To our knowledge, this is the first study to characterize the changes in Ca^2+^-related factors in response to repeated electromyostimulation and the first to show the time course for these changes, with peak SLN expression occurring after the 5th session.

### 4.1 Sarcolipin and SERCA expression

SLN expression was significantly higher after the 5th and 10th sessions of repeated electromyostimulation, whereas SERCA expression remained unchanged. Previous studies have shown that SLN expression in mouse and human skeletal muscles increases with chronic mechanical stress [[22], [23], [24]], and our findings support these results. Our results also align with previous studies showing that resistance training does not alter SERCA expression [33,34]. SLN activates Ca^2+^ signaling by elevating cytosolic Ca^2+^ concentration when SLN binds to and inhibits SERCA [[16], [17], [18], [19]]. Therefore, the increased SLN expression observed here may have altered intracellular Ca^2+^ metabolism by inhibiting SERCA activity [[16], [17], [18], [19]].

### 4.2 Sarcolipin and Ca^2+^ signaling factors

We investigated the relationship between SLN expression and Ca^2+^ signaling factors after repeated resistance exercise. We found significant positive correlations between SLN and RCAN1 and between SLN and CaMKII γ expression and a trend towards positive correlation between SLN and CaMKII expression. RCAN1 expression is known to be induced by calcineurin activation, making it an indirect marker of calcineurin activity [35]. Previous studies have reported that SLN expression influences calcineurin and CaMKII signaling pathways [8,13,24], which aligns with the present findings. Thus, our results suggest that SLN expression is also related to activation of Ca^2+^ signaling after exercise. The activation of calcineurin and CaMKII is triggered by increased cytosolic Ca^2+^ concentration [11]. A previous study reported that the inhibitory effect of SLN on SERCA was greater during the 1st muscle contraction than the 10th muscle contraction [36]. Therefore, increased SLN expression induced by repeated electromyostimulation may place a greater demand on Ca^2+^ metabolism, particularly during the initial contractions of a set, and contribute to enhanced Ca^2+^ signaling. However, the specific changes in Ca^2+^ concentration caused by increased SLN expression will need to be clarified through real-time observation in future studies.

Interestingly, among the 3 CaMKII isoforms measured, only CaMKII γ showed a significant positive correlation with SLN expression. Previous studies have reported that CaMKII activation varies depending on the isoform [[37], [38], [39]] and that differences in resistance exercise load may differentially affect isoform expression [22]. However, the specific roles of each isoform in activation remain unclear [22,40]. Therefore, the observed association between SLN and CaMKII γ warrants further investigation. Notably, there was no significant main effect of exercise in the activation of CaMKII or its isoforms. One possible reason for this is that the non-stimulated legs of the same animal were used as the control. Since CaMKII acts as a decoder of changes in Ca^2+^ concentration [11], the present results suggest that Ca^2+^ concentration also increased in the control legs. Although electromyostimulation was performed on only one leg, changes in the rats’ daily activity patterns cannot be ruled out. Thus, future studies may need to use separate animals as controls when assessing changes in Ca^2+^ signaling factors.

### 4.3 Sarcolipin regulatory factors

SLN expression was significantly higher after the 5th and 10th sessions, and calcineurin expression showed a significant main effect of exercise. Previous studies have reported that calcineurin has a positive feedback loop, being activated by SLN-induced increases in cytosolic Ca^2+^ concentration and subsequently promoting SLN expression [13,20,24]. RCAN1 expression, which is induced by calcineurin activation [35], was also significantly higher after the 5th and 10th sessions. These results suggest that electromyostimulation may promote SLN expression by increasing both calcineurin expression and activation.

However, SLN expression in exercised legs did not increase between the 5th and 10th sessions. This may be related to the observed change in RCAN1 expression. RCAN1 expression suppresses SLN expression by suppressing calcineurin expression [20,35]. Accordingly, the plateau in SLN expression may be explained by increased RCAN1 expression in the 10th session compared with the 5th. SLN has been reported to be excessively expressed in pathological conditions such as muscular dystrophy [41,42]. This suggests that a mechanism may be at work to prevent excessive increases in SLN expression induced by resistance exercise, by increasing RCAN1 expression levels. Together, these findings imply that repeated electromyostimulation may enhance SLN expression by upregulating calcineurin, while also exerting a suppressive effect through a progressive increase in RCAN1 expression.

SLN has been suggested to be effective in improving endurance performance [28]. SLN is also known to be an important factor in non-shivering thermogenesis in skeletal muscle, and is attracting attention as a potential target factor for obesity prevention [43,44]. Therefore, the results of this study provide fundamental knowledge that will lead to the development of new training methods for humans that focus on SLN expression levels.

## 5 Limitations

Although we observed a positive correlation between SLN and several Ca^2+^ signaling factors throughout the training period, it remains unclear whether exercise-induced changes in SLN expression directly influence intracellular Ca^2+^ concentration or the expression of Ca^2+^ signaling proteins. Thus, future studies will need to determine the direct effects of SLN on exercise-induced changes in Ca^2+^ signaling using models such as SLN overexpression or knockout.

## 6 Conclusion

Repeated electromyostimulation to simulate resistance exercise increased SLN expression in rat skeletal muscles. SLN expression correlated with some Ca^2+^ signaling factors, suggesting that increased SLN expression induced by electromyostimulation may contribute to enhanced Ca^2+^ signaling after exercise.

## Funding

This work was supported by 10.13039/501100001691JSPS KAKENHI [grant number 17H02183] granted to S.F. and [grant number 19K19963] granted to J.T.

## CRediT authorship contribution statement

**Koki Okumura:** Conceptualization, Formal analysis, Validation, Writing – original draft, Writing – review & editing. **Junya Takegaki:** Conceptualization, Methodology, Validation, Writing – review & editing. **Kohei Sase:** Methodology, Validation, Writing – review & editing. **Naoki Fukao:** Conceptualization, Validation, Writing – review & editing. **Satoshi Fujita:** Conceptualization, Supervision, Validation, Writing – review & editing.

## Declaration of competing interest

The authors declare no competing interests.

## Data Availability

Data will be made available on request.

## References

[1] Bandy W.D., Lovelace-Chandler V., McKitrick-Bandy B. (1990). Adaptation of skeletal muscle to resistance training. J. Orthop. Sports Phys. Ther..

[2] Westcott W.L. (2012). Resistance training is medicine: effects of strength training on health. Curr. Sports Med. Rep..

[3] Groennebaek T., Vissing K. (2017). Impact of resistance training on skeletal muscle mitochondrial biogenesis, content, and function. Front. Physiol..

[4] McLeod J.C., Currier B.S., Lowisz C.V., Phillips S.M. (2024). The influence of resistance exercise training prescription variables on skeletal muscle mass, strength, and physical function in healthy adults: an umbrella review. J. Sport. Health Sci..

[5] Chin E.R., Allen D.G. (1997). Effects of reduced muscle glycogen concentration on force, Ca^2+^ release and contractile protein function in intact mouse skeletal muscle. J. Physiol..

[6] Olsson K., Cheng A.J., Al-Ameri M., Wyckelsma V.L., Rullman E., Westerblad H., Lanner J.T., Gustafsson T., Bruton J.D. (2020). Impaired sarcoplasmic reticulum Ca^2+^ release is the major cause of fatigue-induced force loss in intact single fibres from human intercostal muscle. J. Physiol..

[7] Ito N., Ruegg U.T., Kudo A., Miyagoe-Suzuki Y., Takeda S. (2013). Activation of calcium signaling through Trpv1 by nNOS and peroxynitrite as a key trigger of skeletal muscle hypertrophy. Nat. Med..

[8] Maurya S.K., Herrera J.L., Sahoo S.K., Reis F.C.G., Vega R.B., Kelly D.P., Periasamy M. (2018). Sarcolipin signaling promotes mitochondrial biogenesis and oxidative metabolism in skeletal muscle. Cell Rep..

[9] Joseph J.S., Anand K., Malindisa S.T., Oladipo A.O., Fagbohun O.F. (2021). Exercise, CaMKII, and type 2 diabetes. EXCLI J..

[10] Richter E.A., Hargreaves M. (2013). Exercise, GLUT4, and skeletal muscle glucose uptake. Physiol. Rev..

[11] Tavi P., Westerblad H. (2011). The role of in vivo Ca^2+^ signals acting on Ca^2+^ calmodulin-dependent proteins for skeletal muscle plasticity. J. Physiol..

[12] Blaauw B., Schiaffino S., Reggiani C. (2013). Mechanisms modulating skeletal muscle phenotype. Compr. Physiol..

[13] Maurya S.K., Bal N.C., Sopariwala D.H., Pant M., Rowland L.A., Shaikh S.A., Periasamy M. (2015). Sarcolipin is a key determinant of the basal metabolic rate, and its overexpression enhances energy expenditure and resistance against diet-induced obesity. J. Biol. Chem..

[14] Chin E.R., Olson E.N., Richardson J.A., Yang Q., Humphries C., Shelton J.M., Wu H., Zhu W., Bassel-Duby R., Williams R.S. (1998). A calcineurin-dependent transcriptional pathway controls skeletal muscle fiber type. Genes Dev..

[15] Fukutani A., Westerblad H., Jardemark K., Bruton J. (2024). Ca^2+^ and force during dynamic contractions in mouse intact skeletal muscle fibers. Sci. Rep..

[16] Odermatt A., Becker S., Khanna V.K., Kurzydlowski K., Leisner E., Pette D., MacLennan D.H. (1998). Sarcolipin regulates the activity of SERCA1, the fast-twitch skeletal muscle sarcoplasmic reticulum Ca^2+^-ATPase. J. Biol. Chem..

[17] Fajardo V.A., Bombardier E., Vigna C., Devji T., Bloemberg D., Gamu D., Gramolini A.O., Quadrilatero J., Tupling A.R. (2013). Co-expression of SERCA isoforms, phospholamban and sarcolipin in human skeletal muscle fibers. PLoS One.

[18] Sahoo S.K., Shaikh S.A., Sopariwala D.H., Bal N.C., Periasamy M. (2013). Sarcolipin protein interaction with sarco(endo)plasmic reticulum Ca^2+^ ATPase (SERCA) is distinct from phospholamban protein, and only sarcolipin can promote uncoupling of the SERCA pump. J. Biol. Chem..

[19] Sahoo S.K., Shaikh S.A., Sopariwala D.H., Bal N.C., Bruhn D.S., Kopec W., Khandelia H., Periasamy M. (2015). The N terminus of sarcolipin plays an important role in uncoupling sarco-endoplasmic reticulum Ca^2+^-ATPase (SERCA) ATP hydrolysis from Ca^2+^ transport. J. Biol. Chem..

[20] Rotter D., Peiris H., Grinsfelder D.B., Martin A.M., Burchfield J., Parra V., Hull C., Morales C.R., Jessup C.F., Matusica D., Parks B.W., Lusis A.J., Nguyen N.U.N., Oh M., Iyoke I., Jakkampudi T., McMillan D.R., Sadek H.A., Watt M.J., Gupta R.K., Pritchard M.A., Keating D.J., Rothermel B.A. (2018). Regulator of Calcineurin 1 helps coordinate whole-body metabolism and thermogenesis. EMBO Rep..

[21] Fuentes J.J., Genesca L., Kingsbury T.J., Cunningham K.W., Perez-Riba M., Estivill X., de la Luna S. (2000). DSCR1, overexpressed in Down syndrome, is an inhibitor of calcineurin-mediated signaling pathways. Hum. Mol. Genet..

[22] Martinez-Canton M., Gallego-Selles A., Gelabert-Rebato M., Martin-Rincon M., Pareja-Blanco F., Rodriguez-Rosell D., Morales-Alamo D., Sanchis-Moysi J., Dorado C., Jose Gonzalez-Badillo J., Calbet J.A.L. (2021). Role of CaMKII and sarcolipin in muscle adaptations to strength training with different levels of fatigue in the set. Scand. J. Med. Sci. Sports.

[23] Riedl I., Osler M.E., Björnholm M., Egan B., Nader G.A., Chibalin A.V., Zierath J.R. (2016). AMPKγ3 is dispensable for skeletal muscle hypertrophy induced by functional overload. Am. J. Physiol. Endocrinol. Metab..

[24] Fajardo V.A., Rietze B.A., Chambers P.J., Bellissimo C., Bombardier E., Quadrilatero J., Tupling A.R. (2017). Effects of sarcolipin deletion on skeletal muscle adaptive responses to functional overload and unload. Am. J. Physiol. Cell Physiol..

[25] Son J.S., Chae S.A., Zhao L., Wang H., de Avila J.M., Zhu M.J., Jiang Z., Du M. (2022). Maternal exercise intergenerationally drives muscle-based thermogenesis via activation of apelin-AMPK signaling. EBioMedicine.

[26] Bal N.C., Maurya S.K., Sopariwala D.H., Sahoo S.K., Gupta S.C., Shaikh S.A., Pant M., Rowland L.A., Bombardier E., Goonasekera S.A., Tupling A.R., Molkentin J.D., Periasamy M. (2012). Sarcolipin is a newly identified regulator of muscle-based thermogenesis in mammals. Nat. Med..

[27] Paran C.W., Verkerke A.R., Heden T.D., Park S., Zou K., Lawson H.A., Song H., Turk J., Houmard J.A., Funai K. (2015). Reduced efficiency of sarcolipin-dependent respiration in myocytes from humans with severe obesity. Obesity.

[28] Sopariwala D.H., Pant M., Shaikh S.A., Goonasekera S.A., Molkentin J.D., Weisleder N., Ma J., Pan Z., Periasamy M. (1985). Sarcolipin overexpression improves muscle energetics and reduces fatigue. J. Appl. Physiol..

[29] Ogasawara R., Sato K., Matsutani K., Nakazato K., Fujita S. (2014). The order of concurrent endurance and resistance exercise modifies mTOR signaling and protein synthesis in rat skeletal muscle. Am. J. Physiol. Endocrinol. Metab..

[30] Takegaki J., Sase K., Fujita S. (2019). Repeated bouts of resistance exercise attenuate mitogen-activated protein-kinase signal responses in rat skeletal muscle. Biochem. Biophys. Res. Commun..

[31] Ogasawara R., Fujita S., Hornberger T.A., Kitaoka Y., Makanae Y., Nakazato K., Naokata I. (2016). The role of mTOR signalling in the regulation of skeletal muscle mass in a rodent model of resistance exercise. Sci. Rep..

[32] Takegaki J., Sase K., Kono Y., Nakano D., Fujita T., Konishi S., Fujita S. (2021). Intramuscular injection of mesenchymal stem cells activates anabolic and catabolic systems in mouse skeletal muscle. Sci. Rep..

[33] Tøien T., Haglo H., Nyberg S.K., Rao S.V., Stunes A.K., Mosti M.P., Wang E. (2021). Maximal strength training-induced increase in efferent neural drive is not reflected in relative protein expression of SERCA. Eur. J. Appl. Physiol..

[34] Łochyński D., Kaczmarek D., Grześkowiak M., Majerczak J., Podgórski T., Celichowski J. (2021). Motor unit force potentiation and calcium handling protein concentration in rat fast muscle after resistance training. Front. Physiol..

[35] Yang J., Rothermel B., Vega R.B., Frey N., McKinsey T.A., Olson E.N., Bassel-Duby R., Williams R.S. (2000). Independent signals control expression of the calcineurin inhibitory proteins MCIP1 and MCIP2 in striated muscles. Circ. Res..

[36] Tupling A.R., Bombardier E., Gupta S.C., Hussain D., Vigna C., Bloemberg D., Quadrilatero J., Trivieri M.G., Babu G.J., Backx P.H., Periasamy M., MacLennan D.H., Gramolini A.O. (2011). Enhanced Ca^2+^ transport and muscle relaxation in skeletal muscle from sarcolipin-null mice. Am. J. Physiol. Cell Physiol..

[37] De Koninck P., Schulman H. (1998). Sensitivity of CaM kinase II to the frequency of Ca^2+^ oscillations. Science.

[38] Gaertner T.R., Kolodziej S.J., Wang D., Kobayashi R., Koomen J.M., Stoops J.K., Waxham M.N. (2004). Comparative analyses of the three-dimensional structures and enzymatic properties of alpha, beta, gamma and delta isoforms of Ca^2+^-calmodulin-dependent protein kinase II. J. Biol. Chem..

[39] Eilers W., Gevers W., van Overbeek D., de Haan A., Jaspers R.T., Hilbers P.A., van Riel N., Flück M. (2014). Muscle-type specific autophosphorylation of CaMKII isoforms after paced contractions. BioMed Res. Int..

[40] Chin E.R. (1985). Role of Ca^2+^/calmodulin-dependent kinases in skeletal muscle plasticity. J. Appl. Physiol..

[41] Fajardo V.A., Chambers P.J., Juracic E.S., Rietze B.A., Gamu D., Bellissimo C., Kwon F., Quadrilatero J., Russell Tupling A. (2018). Sarcolipin deletion in mdx mice impairs calcineurin signalling and worsens dystrophic pathology. Hum. Mol. Genet..

[42] Wang W.P., Wang J.Y., Lin W.H., Kao C.H., Hung M.C., Teng Y.C., Tsai T.F., Chi Y.H. (2020). Progerin in muscle leads to thermogenic and metabolic defects via impaired calcium homeostasis. Aging Cell.

[43] Maurya S.K., Periasamy M. (2015). Sarcolipin is a novel regulator of muscle metabolism and obesity. Pharmacol. Res..

[44] Bal N.C., Periasamy M. (2020). Uncoupling of sarcoendoplasmic reticulum calcium ATPase pump activity by sarcolipin as the basis for muscle non-shivering thermogenesis. Philos. Trans. R. Soc. Lond. B Biol. Sci..

